# Procrustes analysis of a shape of pediatric supracondylar humerus fractures

**DOI:** 10.1038/s41598-024-64347-3

**Published:** 2024-06-10

**Authors:** Ryszard Tomaszewski, Jerzy Dajka

**Affiliations:** 1 Department of Pediatric Orthopedics and Traumatology, Medical University of Silesia, 40-752 Katowice, Poland; 2 Institute of Biomedical Engineering, University of Silesia in Katowice, 40-007 Katowice, Poland; 3 Institute of Physics, University of Silesia in Katowice, 40-007 Katowice, Poland; 4 The Professor Tadeusz Widła Interdisciplinary Research Centre for Forensic Science and Legislation, University of Silesia in Katowice, Katowice, Poland

**Keywords:** Supracondylar humeral fractures, Children, Statistical image analysis, Procrustes method, Paediatric research, Computational biophysics, Paediatric research, Bone imaging, Translational research, Biomedical engineering, Statistics

## Abstract

Shape of supracondylar fracture of the humeral of pediatric patients is analysed with Procrustes method. XR-images of fractures are considered both in anterio-posterior (AP) view and in a lateral (L) view. Applying Procrustes method for both views mean images are constructed and compared. Variability of shapes is quantified with a shape principal component analysis. Possibility of predictions of typical shape of humeral fracture and its variability using statistical shape analysis offers additional information on injury characteristics important in preoperative planning. Non-parametric tests (permutational and bootstrap) do not indicate statistical difference between Procrustes mean shapes in anterio-posterior and lateral projections. It is shown, however, that AP and L shapes of humeral fractures differ in their variability quantified by shape principal components.

## Introduction

The ongoing advancements in technology and theoretical methodologies have propelled medical imaging to become an essential tool in contemporary medicine^1,2^. Among these imaging techniques, plain radiography, despite its simplicity and long-standing history, continues to hold a prominent position in the diagnosis and treatment of bone fractures, making it an indispensable preliminary diagnostic modality. Plain radiographs are particularly valuable in the context of surgical interventions as they facilitate basic preoperative planning, including the classification of bone fractures and the determination of appropriate treatment approaches^3^. Consequently, radiography has gained significant attention within the field of traumatology^4^.

The supracondylar fracture of the humeral stands as the predominant injury in the pediatric population^5^, constituting approximately 16% of all fractures in children^6–8^. Furthermore, it accounts for more than 60% of all elbow fractures among children^9–11^. The primary mechanism of this fracture type typically involves an extension-type injury, which transpires when a child experiences a fall on an outstretched hand, with the elbow fully extended and an abduction occurring at the shoulder joint^12^. Remarkably, this specific subtype of supracondylar fracture constitutes a significant majority, representing approximately 97–99% of all cases^9,13–16^. In certain circumstances, the morphological characteristics of the injury displayed in medical images become of particular interest^17,18^.

Gartland classification is most often used in qualifying patients for conservative or surgical treatment of supracondylar humeral fractures. This classification is based on the evaluation of the displacement of the fracture of the distal end of the humerus in lateral projection. However, in the treatment of mainly surgical fractures, the descriptive assessment of the shape of the fracture based on X-ray images is of primary importance. The shape of a fracture associated with an injury can serve as a valuable example. Understanding the specific characteristics of the fracture shape not only helps to deduce the mechanism by which the injury occurred but also aids in treatment planning^19^. This inherent interest in studying fracture shapes stems from the natural desire to gain insights into injury mechanisms and optimize treatment strategies. Additionally, the growing importance of preoperative modeling for bone reconstruction further emphasizes the significance of fracture shape analysis^3^. Recognizing that every image represents a unique set of data, there is an ongoing endeavor to enhance specialists’ knowledge and expertise through the utilization of automated computer-based tools. These tools predominantly emerge from the data science community, aiming to provide support and augment the capabilities of medical professionals. The application of statistical shape analysis in orthopedic problems^4,20–23^ is driven by a compelling realization. On one hand, the shapes of fractures observed in radiological images originate from the same population. However, on the other hand, these shapes exhibit variations that are subject-specific, posing a challenge in accurately describing shape variability. To address this challenge, one of the objectives, formalized within the framework of statistics^24,25^, is to construct a mean shape characterized by population-averaged parameters. Subsequently, the subject-specific variation of this mean shape can be investigated by considering random effects. A key characteristic of shape is its invariance to translation, rotation, and size transformations. To remove irrelevant shape features, the Procrustes model^26^ has been proposed, leading to the development of Procrustes analysis, which involves matching configurations based on similarity measures.

Our aim was to investigate the *typical* shape of a supracondylar fracture in pediatric patients. We limited our considerations to a well-defined but common type of injury to construct the mean shape of the fracture as observed in radiographic imaging from both anterio-posterior (AP) and lateral (L) perspectives. As a primary tool, we used the Procrustes method to extract the shape, i.e., “all the geometrical information that remains when location, scale, and rotational effects are removed from an object”^27^. Since the most common radiographic images are two-dimensional, we investigated whether the mean (typical) shape of the supracondylar fracture depended on the perspective, i.e., whether it was statistically different in AP and L perspectives. We further extended the statistical analysis of supracondylar humerus fractures to study the variability of the shapes encoded in the principal components of the shape covariance matrix. Our objective was to provide results on typical shapes of a class of fractures, which in principle serve as auxiliary information for preoperative planning.

Our paper is organized as follows: in “Materials and methods” section we describe how our data, primarily stored in radiographic images, become digitalized into landmark points and we briefly review both statistical methods and algorithms applied for the Procrustes shape analysis. In further sections we report and discuss results of our investigation.

## Materials and methods

We conducted a study involving 66 radiographic (XR) images captured in the anterio-posterior (AP) view and 24 images taken in the lateral (L) view of supracondylar fractures of the humeral in pediatric patients who were admitted to the Pediatric Trauma-Orthopedic Department. All the methods were performed in accordance with the relevant guidelines and regulations. In the AP image group, there were 46 boys and 20 girls, the average age of patients was 8.2 years (3–11); and in the lateral group, there were 14 boys and 10 girls, the average age of patients was 8.6 years (3–11).

Our analysis consists of three stages: fracture image digitization, determination of mean shapes of fractures in both AP and L perspective and, using Principal Component Analysis, quantification of shape variability.

### Fracture digitization

**Figure 1:**
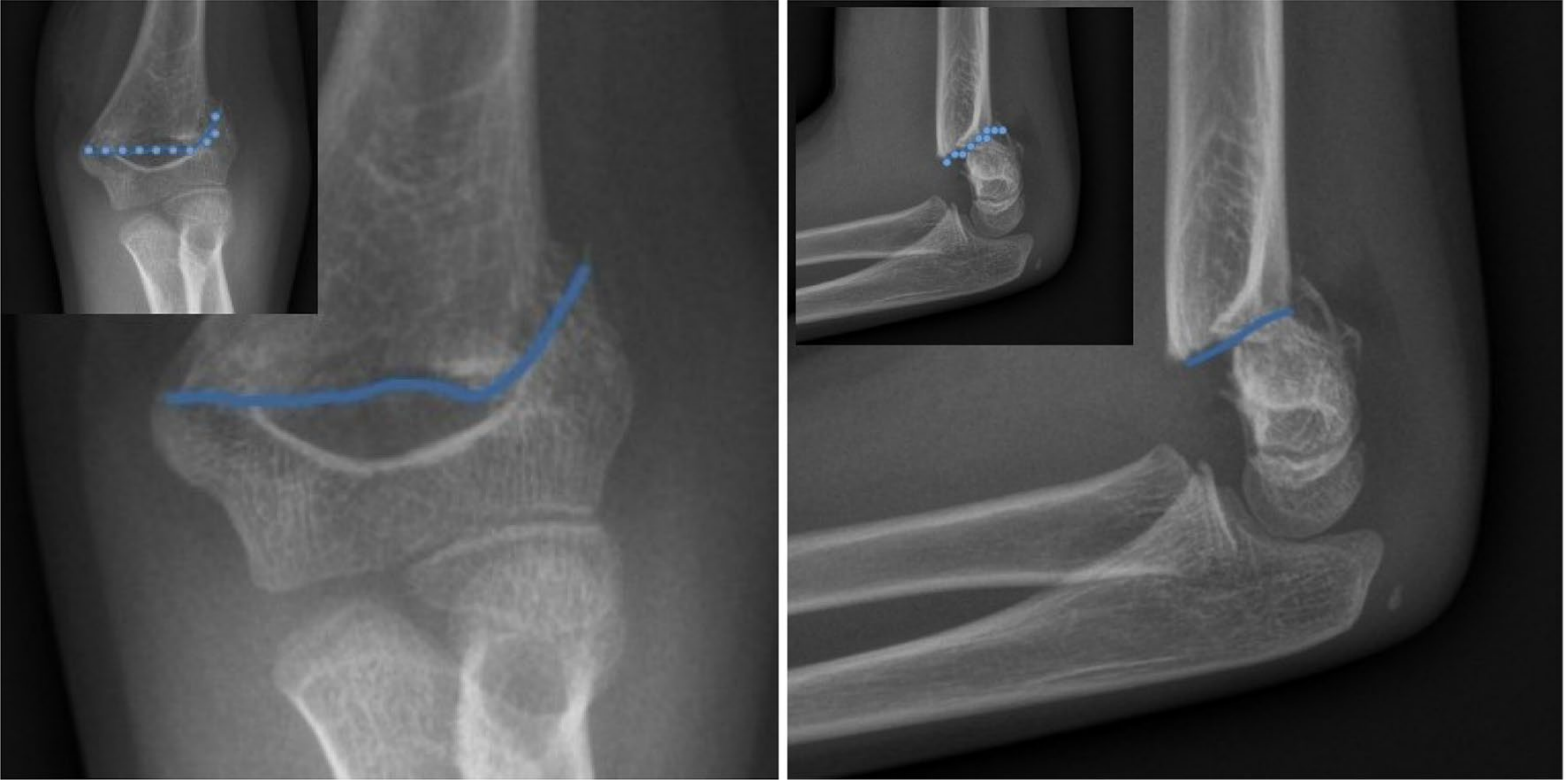
Examples of XR images of humerus fractures: front (AP) and side (L) projections with fracture shape approximated from uniformly distributed 10 landmarks. Insets present landmarks used for fracture digitization cf. Materials and Methods for details.

To analyze the shapes of fractures encoded in the XR images we employed Procrustes analysis, which allowed us to eliminate irrelevant shape features. Each fracture was discertized using a set of 10 scientific landmarks that were assigned by experts cf. Fig. 1. This approach of using a relatively large number of landmarks helps to mitigate potential biases that may arise from an expert’s subjective selection of specific landmarks. Consequently, our strategy ensures a quasi-random and uniform selection of landmarks.

To ensure the utilization of open-source and freely available computational tools, we made a deliberate choice for our study. The initial preparation of images was conducted using the Python ’skimage’ package. Scientific landmarks for each fracture were extracted from the XR images, as shown in Fig. 1, using the ’FigureCanvasBase.mpl-connect’ method from the Matplotlib library. The landmarks were further encoded into a configuration matrix *X* invariant under location, rotation, and isotropic scaling (Euclidean similarity transformations)^27^. In formal terms, a shape, denoted as *X*, belongs to a shape space M, which is a subset of the real Cartesian product, i.e., $X\in M\subset {\textbf{R}}^{10\times 2}$, where $k = 10$ represents the number of landmarks in each 2-dimensional image^27^. With *a*, *b* labelling observers, the intra-observer agreement on a selection of landmarks of the particular *i*-th image $X_i^a,X_i^b$ was assumed to be granted provided that distance (Riemannian shape distance $\rho$^27^) between curves reconstructed from $X_i^a,X_i^b$ is smaller than $5\%$ of mean fracture length i.e.

1 $$\begin{aligned} \frac{2\rho (X_i^a,X_i)^b}{l_a+l_b} < 0.05 \end{aligned}$$

where, respectively, $i=1,\ldots ,66$ for the AP-view ($i=1,\ldots ,24$ for the L-view) of fracture shapes and $l_k, k=a,b$ denotes length of the fracture shape (approximation of the fracture) constructed upon experts’ landmarks $X_i^a,X_i^b$. To calculate $\rho$ we use ’riemdist’ function from the R-package ’shapes’^28^. Resulting configuration matrices are collected and accessible cf. Additional information below.

### Determination of typical shape and its variability

After digitizing the shape of a fracture using scientific landmarks our objective was to construct and analyze the mean shape for both the anterio-posterior (AP) and lateral (L) projections. Procrustes matching^19,29,30^, as summarized in Ref.^27^ and applied in this study, involves registering all shapes (fractures) to their optimal positions by applying translation, rotation, and rescaling operations. This objective was achieved by minimizing the sum of squared Euclidean distances between the shapes. By doing so, we estimated the mean shape denoted as *E*[*X*] or the Procrustes mean shape^27^. Estimating the mean shape was complemented by determining the structure of shape variability. Average shape of the fracture obtained via the Procrustes method is robust with respect to minor imperfections in both patient’s positioning during XR imaging^31^ and imprecise designing of scientific landmarks. Moreover, it is not affected by an image size. The very essence of Procrustes method is to minimize a total sum of squares

2 $$\begin{aligned} \sum _{i=1}^n||\beta _i X_i\Gamma _i+ {\mathbb {I}}\gamma _i-\mu ||\rightarrow \text{ min. } \end{aligned}$$

where the Procrustes mean is calculated as a mean value of Procrustes coordinates $X_i^P$ as follows:

3 $$\begin{aligned} E[X]={\bar{X}}=\frac{1}{n}\sum _{i=1}^n X_i^P=\frac{1}{n}\sum _{i=1}^n(\beta _i X_i\Gamma _i +{\mathbb {I}}\gamma _i) \end{aligned}$$

In calculations we apply ’procGPA’ function available in the “shapes” R-package^28^. It implements a three-stage algorithm consisting of the following steps: *(i)*. Translations $X_i^P=CX_i$ where *C* describes centering of coordinates. *(ii)*. Rotations of the *i*th configuration ${\bar{X}}_i=\frac{1}{n-1}\sum _{j\ne i}X_j^P$ leading to a new Procrustes registration $X^P_i$ involving only rotation of the old $X^P_i$. The *n* figures are rotated in turn. *(iii)* Scalling $\beta _i=\left( \frac{\sum _{k=1}^n||X_k^P||^2}{|X_i^P||^2}\right) ^{1/2}\phi _i,$ where $[\phi _i]_{i=1}^n$ is an eigenvector of the correlation matrix obtained from verctorizing $X_i^P$. Steps *(ii)* and *(iii)* are repeated until the Procrustes sum of squares of Eq. (2) is less than a tolerance parameters tol1, tol2 used further by ’procGPA’ function. Further both the tolerance for optimal rotation tol1 and the tolerance for rescalling step tol2 are always set tol1$=$tol2$=10^{-5}$.

Procurustes mean shapes are plotted using cubic spline interpolation. We utilize the ’interpolate’ module from the SciPy package. Statistical comparisons of mean shapes between the AP and L views is performed using non-parametric methods, employing permutations and bootstrapping techniques through the ’resampletest’ function available in the “shapes” R-package allowing to tests mean shape difference using complex arithmetic applicable in two dimensions. To quantify significance of the difference in shapes we used p-value of the Hotelling test included im the ’resampletest’ function.

In statistical shape theory a probabilistic ’events’ (the shapes), even in a planar case considered here, are elements of a high-dimensional space demanding multivariate methods. Standard multivariate parametric methods (such as MANOVA or complex-valued linear modelling) in statistical fracture shape analysis were not suitable as our data fail to satisfy multivariate normality. To verify normality we used the Mardia’s test available in the “QuantPsyc” R-package.

To provide a comprehensive description of the humeral fracture shapes, it is important to quantify their variability. A standard method for analyzing shape variability is Principal Component Analysis (PCA) to identify the most significant features influencing the covariance matrix of the shapes. A suitable measure for this purpose is the population covariance matrix of the coordinates calculated in the tangent space^27^. This tangent space, denoted as $T_{\mu }(M)$, is constructed for the shape space *M* with the pole at the projection $\mu$, which represents the mean shape. In other words, the tangent space is the space tangent to the manifold *M* at the mean shape. The population covariance matrix

4 $$\begin{aligned} \Sigma = E[(V-E[V])(V-E[V])^T] \end{aligned}$$

(where $V\in T_{\mu }(M)$) captures the variability of shapes around the mean shape and provides valuable information about the shape distribution. It describes how shapes deviate from the mean shape in different directions and magnitudes. The calculation of the population covariance matrix in the tangent space is based on the landmarks’ coordinates of the shapes in the dataset. The PCA analysis was conducted using the ’shapepca’ function from the aforementioned “shapes” R-package^27,28^.

### Ethical approval

(PCN/022/KB/11/21) was waived by the local Ethics Committee of the Silesian Medical University of Katowice, Poland, because of the retrospective nature of the study and the fact that the procedures were part of routine care and in informed consent from all subjects and/or their legal guardian(s) was waived for the study.

## Results

### Mean shape of humeral fracture

**Figure 2:**
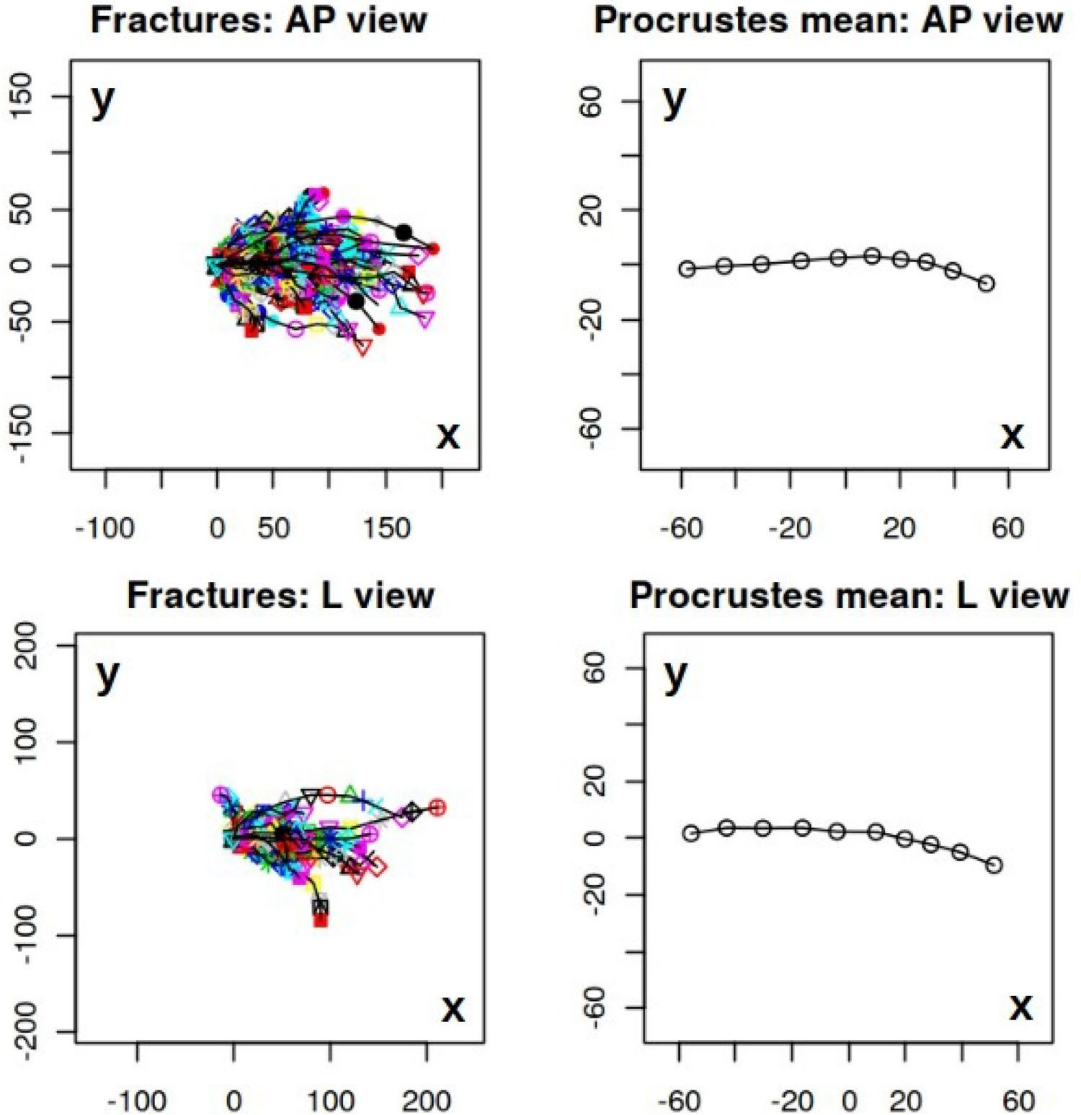
Fracture shapes (sample set) constructed upon 10 landmarks approximation (left panels) and the corresponding Procrustes means (right panels) for the anterio-posterior (AP) and lateral (L) projections. The shapes on the left panels are translated to start at the same point. As the Procrustes method for mean shape estimation is size-insensitive the units in both *x* and *y* directions are arbitrary.

**Figure 3:**
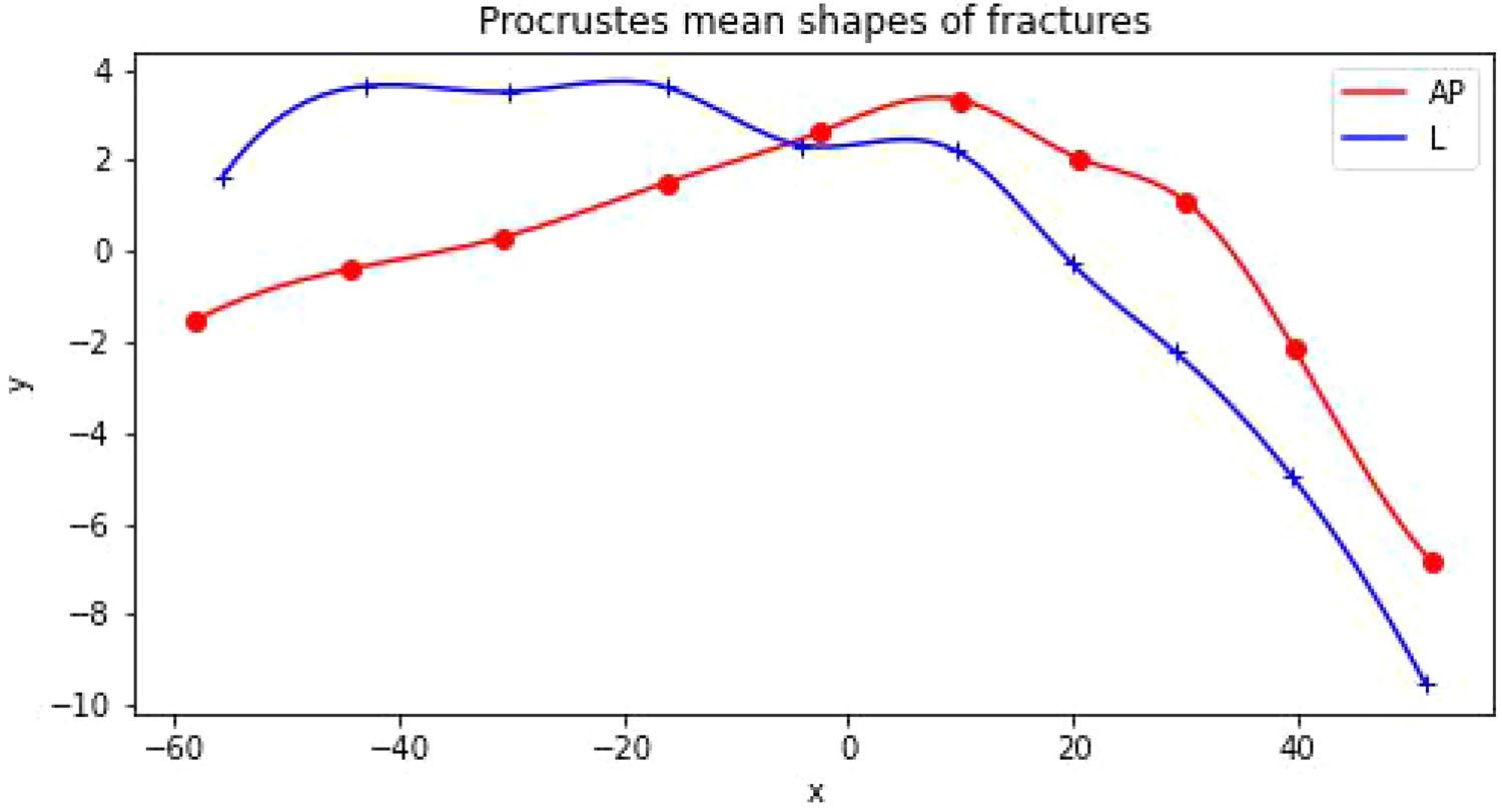
Procrustes mean shapes of humerus fractures in anterio-posterior (AP) and lateral (L) views interpolated with cubic splines. As the Procrustes method for mean shape estimation is size-insensitive the units in both *x* and *y* directions are arbitrary.

Our initial objective was to construct a mean (typical) shape for humeral fractures in both the anterio-posterior (AP) and lateral (L) views. By identifying and selecting k = 10 scientific landmarks from each XR image as shown in Fig. 1, cf. Additional information below, we constructed a sample set of shapes, presented in the left panels of Fig. 2, for both the AP and L views. The observed differences in shapes, as shown in Fig. 2, arise not only from the distinct characteristics of the corresponding fractures but also from technical factors and circumstances related to the radiographic imaging process. These differences can be attributed to variations in factors such as patient age or minor positional variations during the radiography procedure. However, it is crucial to note that all unwanted and artificial features in the analyzed XR images become irrelevant and can be disregarded with the application of the Procrustes method, as employed in this study, to construct the mean fracture shapes. The Procrustes mean shapes, derived from the analysis, are presented in the right panels of Fig. 2. The mean shapes of fractures in the AP and L views are compared in Fig. 3. To enhance the visualization and facilitate further analysis, the Procrustes mean shapes are accompanied by cubic spline interpolation. This interpolation technique ensures mathematical continuity and enables the examination and discussion of shape features that require smoothness, such as the convexity of shapes, which can be assessed by analyzing the second derivative. From the observations in Fig. 3, it can be inferred that the convexity of the mean shapes does not depend on the projection (AP or L), with only minor local variations.

To assess the statistical significance of the differences between the mean shapes in the AP and L views, there are specific methods, such as significance tests, that can be employed^27^. These tests allow for the determination of whether the mean shapes in the AP and L views are statistically similar or different. In this particular case, it is unlikely that parametric methods would provide reliable results since the sets of landmarks does not satisfy the normality assumptions. It is supported by examining the p-value for the multivariate Mardia’s test which we applied to verify normality of data. The p-values read as follows: $p<0.05$ for both *AP* and *L* views. In particular for *AP* $p<0.02$ and for *L* $p<0.04$ for both skewness and kurtosis. Therefore normality hypothesis can be rejected and non-parametric counterparts of standard statistical methods are more appropriate and they were applied in our analysis. The non-parametric methods, based on permutations or bootstrap resampling, were used to compare the mean shapes in the AP and L projections. The results obtained from these methods indicate that there is no statistical difference between the mean shapes, i.e., there is no evidence, based solely on p-values ($p>0.43$) of the Hotelling test, to reject the null hypothesis of $\mu _{AP}=\mu _L$.

### Shape variability: principal component analysis

**Figure 4:**
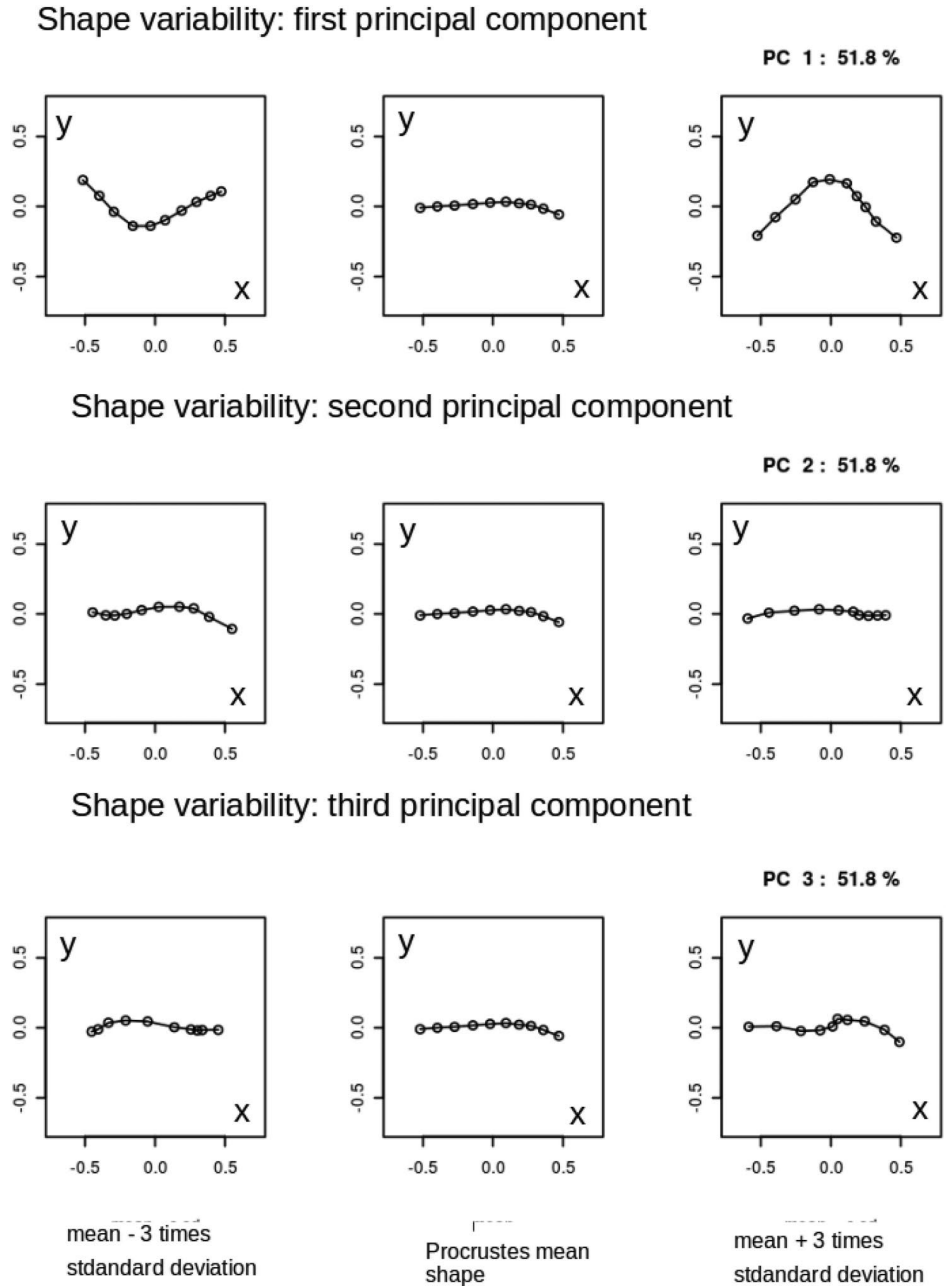
Three principal components of shape variability for the Procrustes mean shape of humerus fractures in anterio-posterior view (AP). Rows of panels correspond to three principal components $j=1,2,3$. In each row row there is Procrustes mean (central panel) presented with a tripled standard deviation subtracted (left column of panels) and added (right column of panels). As the Procrustes method for mean shape estimation is size-insensitive the units in both *x* and *y* directions are arbitrary.

**Figure 5:**
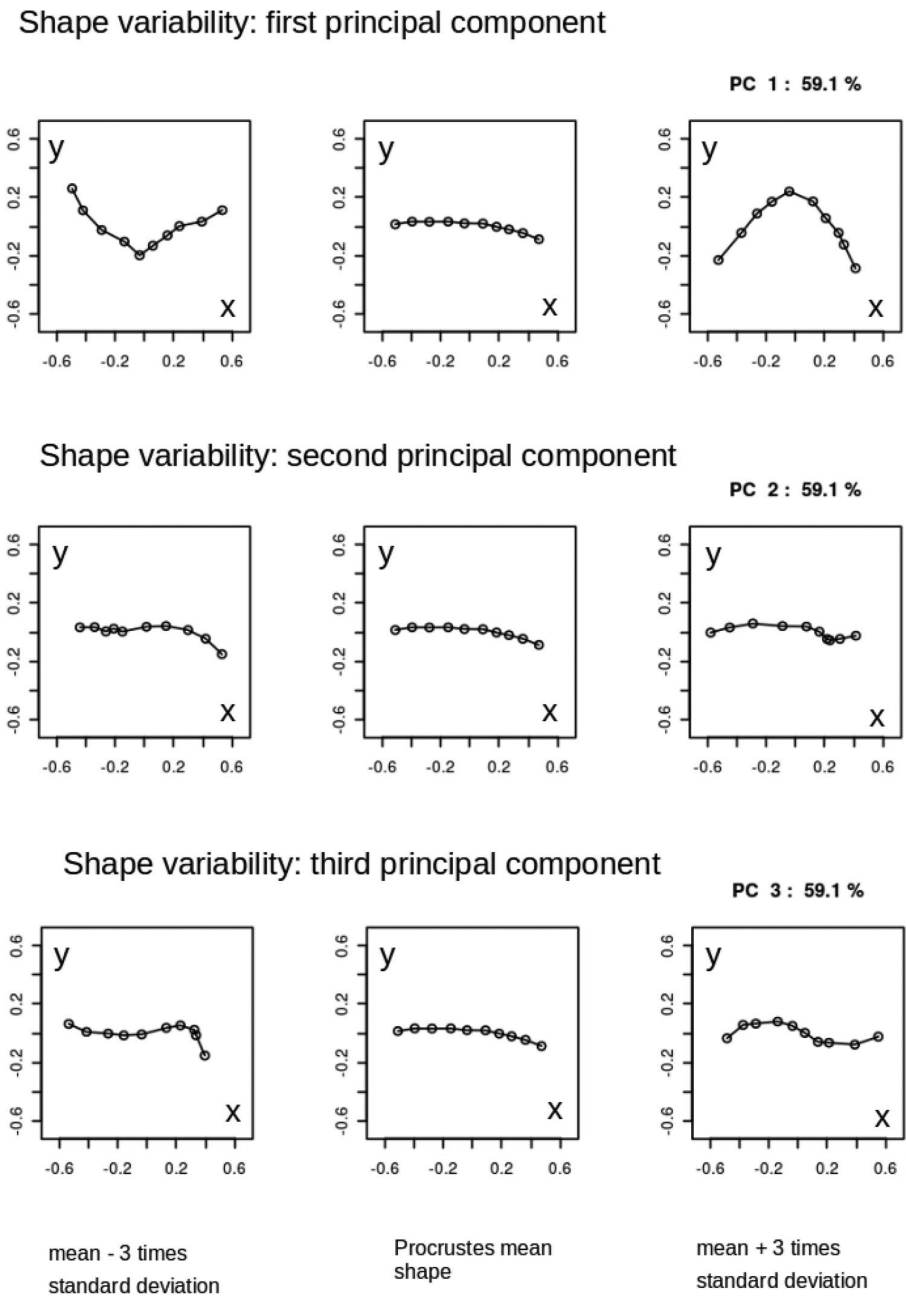
Three principal components of shape variability for the Procrustes mean shape of humerus fractures in lateral view (L). Rows of panels correspond to three principal components $j=1,2,3$. In each row row there is Procrustes mean (central panel) presented with a tripled standard deviation subtracted (left column of panels) and added (right column of panels). As the Procrustes method for mean shape estimation is size-insensitive the units in both *x* and *y* directions are arbitrary.

By analyzing the population covariance matrix, we can gain insights into the patterns and structure of shape variation. This information is crucial for understanding the range of possible shapes within the population and for further statistical analysis and modeling. The results of the Principal Component Analysis (PCA) for the AP and L views are presented in Figs. 4 and 5, respectively. These figures show the principal components of shape variability. It can be observed that for the AP view, the first three principal components account for 59.1%, 11%, and 9.8% of the variability in the mean shape, respectively. For the L view, the corresponding weights are 59.1%, 13%, and 8.8%. Moreover, comparing the principal components between the AP and L views, it can be observed that the lower components (e.g., the second and third) have a stronger impact on the overall variability of the mean shape in the L view. Additionally, there is a qualitative difference in the third principal component between the AP and L views, as indicated by the last row of panels in Figs. 4 and 5.

These results suggest that while there is no statistical difference between the mean shapes in the AP and L projections, there are differences in the principal components that quantify their variability. This finding highlights the importance of considering not only the mean shape but also the higher-order modes of variation when comparing the two projections.

## Discussion

We focused our study on a common pediatric injury known as supracondylar humeral fractures. These fractures pose a significant challenge in the preoperative assessment for surgeons, as the initial and often only available imaging modality is an X-ray image captured in both the anterio-posterior (AP) and lateral (L) projections. These images play a crucial role in determining the appropriate treatment strategy for the patient. By analyzing and interpreting the information provided by both the AP and lateral X-ray images, surgeons can gain insights into the nature and extent of the fracture. This allows them to make informed decisions regarding the surgical approach and plan the necessary interventions for optimal patient outcomes. The combination of information obtained from both projections is vital in developing an effective treatment strategy for supracondylar humeral fractures in pediatric patients.

In our study we utilized Procrustes analysis, a powerful technique described in Ref.^27^, to extract essential features of shape and eliminate irrelevant features such as translation, rotation and size to construct typical (mean) shape of supracondylar humeral fractures for a set 66 anterio-posterior (AP) and 24 lateral (L) radiographic images. Relatively small group of patients and non-equal number of images in AP and L perspective, although not affecting statistical reasoning applied in our study, is a limitation which needs to be emphasised. We showed that the mean shapes in both AP and L projections exhibited similar features, regardless of the projection type. This observation was further supported by statistical analysis using a non-parametric version of the Hotelling’s T-square test, which indicated no significant difference between the mean shapes in the AP and L projections. However, when we examined the variability of the mean shapes, as quantified by the principal components of the covariance matrix, we observed a difference between the AP and L projections. This suggests that further differentiation of supracondylar humeral fracture shapes is possible by considering the principal components of shape variability. In other words the typical or average information regarding the shape of a humeral fracture is consistent for both AP and L imaging. However, it is important to note that further analysis of shape variability reveals noticeable differences between the two views, particularly in the lower principal components of the covariance matrix (Eq. (4)). These differences highlight the distinct variations in shape observed in different views and provide additional insights into the complexity and diversity of humeral fracture shapes. By utilizing the Procrustes method and examining both the mean shapes and the variability of fractures, we gain a comprehensive understanding of the typical properties and variations present in this common injury. This knowledge can be valuable for medical professionals in diagnosing and treating supracondylar humeral fractures in pediatric patients. Almost constant curvature of the typical fracure shape both in AP and L projection may serve as a particular example of such an information of a potential value for preoperative planning. By analyzing and comparing the shapes of fractures, clinicians can gain valuable insights into the biomechanics and patterns of injuries. This knowledge can aid in determining appropriate treatment strategies and optimizing patient outcomes.

The Procrustes method serves as an initial step in the development of more advanced methods for statistical shape analysis. The Procrustes method allows for the alignment and comparison of shapes, which is essential for studying the variability and patterns in shape data. Building upon the Procrustes method, more sophisticated techniques such as mixed models can be employed to analyze shape data. Mixed models take into account the hierarchical structure of the data and can incorporate both fixed effects (such as treatment or imaging orientation) and random effects (such as individual-specific variations). A natural next step in our investigations of typical properties of fractures is to relate them to common methods of classification of injuries such as e.g. Gartland classification or patients age. We hope that our present studies will serve as good starting point contributing both to clinical practice and biomechanical research, providing a deeper understanding of shape variations and their implications.

### Supplementary Information

Supplementary Information.

## Data Availability

*(i)* The raw data (images) that support the findings of this study are available from Department of Pediatric Traumatology and Orthopedy, Upper Silesian Child Centre in Katowice, 40-007 Katowice, Poland, but restrictions apply to the availability of these data, which were used under license for the current study, and so are not publicly available. Data are however available from the authors (Ryszard Tomaszewski) upon reasonable request and with permission of Department of Pediatric Traumatology and Orthopedy, Upper Silesian Child Centre in Katowice, 40-007 Katowice, Poland. *(ii)* The datasets (landmarks of shapes) generated and/or analysed during the current study are available as supplementary information file or in: https://drive.google.com/drive/folders/1TdTrcPnoXigd6PpjGmZHz6ADjbY2q5AT?usp=drive_link.
